# HOXC10 promotes migration and invasion via the WNT-EMT signaling pathway in oral squamous cell carcinoma

**DOI:** 10.7150/jca.30645

**Published:** 2019-07-25

**Authors:** Bo-Wen Dai, Zhi-Min Yang, Ping Deng, Yan-Rong Chen, Zhi-Jing He, Xi Yang, Sheng Zhang, Han-Jiang Wu, Zhen-Hu Ren

**Affiliations:** 1 Department of Oral and Maxillofacial Surgery, The Second Xiangya Hospital of Central South University, Changsha, Hunan, China.; 2 Department of Occupational Health, Third Military Medical University, Chongqing, China; 3 Department of Oral Maxillofacial-Head and Neck Oncology, Ninth People's Hospital, Shanghai Jiao Tong University School of Medicine, Shanghai, China

**Keywords:** HOXC10, WNT10B, oral squamous cell carcinoma, epithelial-mesenchymal transition

## Abstract

As a master regulator of embryonic morphogenesis, homeodomain-containing gene 10 (HOXC10) has been found to promote progression of human cancers and indicate poor survival outcome. Therefore, we concentrate on elucidating the role of HOXC10 in progression of oral squamous cell carcinoma (OSCC). In our study, the expression of HOXC10 was significantly increased in human OSCC samples and was significantly correlated with TNM stage and lymph node metastasis. Upregulation of HOXC10 indicated a poor overall survival of OSCC patients according to the Kaplan-Meier survival curves. Furthermore, HOXC10-knockdown dramatically suppressed migration, invasion, and expression of N-Cadherin, Vimentin and Snail, as well as increased E-cadherin level both *in vivo* and *in vitro*. Bioinformatics and cellular study further confirmed that HOXC10 may promote invasion and migration of OSCC cells by regulating the WNT/epithelial-mesenchymal transition (EMT) signaling pathway. These findings suggest that HOXC10 plays a pivotal role in the metastasis of OSCC and highlight its usefulness as a potential prognostic marker or therapeutic target in human OSCC.

## Introduction

Oral squamous cell carcinoma (OSCC) is a common head and neck cancer with the propensity for local spread and distant metastasis 1. Although the long-term outcome of patients with highgrade OSCC who undergo surgery has been improved by the advent of systemic chemotherapy, the efficacy of most neoadjuvant chemotherapy drugs is uncertain, and these drugs are associated with severe side effects^2^. The outcome for patients remains unfavorable due to molecularly diverse and aberrant signaling pathways in OSCC^3^. Therefore, a better understanding of the OSCC initiation and progression mechanisms is urgently needed.

There are 39 homeobox (HOX) genes organized into four different genomic clusters (HOX A-D) located on four human chromosomes (7, 17, 12, and 2) 4. HOX genes are highly conserved at the genomic level and have been well-described as important players in regulation of numerous processes including apoptosis, receptor signaling, differentiation, motility, angiogenesis and metastasis 5,6. Aberrations in HOX gene expression, which have been reported in numerous malignancies 7,8, significantly enhanced invasiveness, proliferation and colony formation of tumor cells 9,10. HOXC10, as a member of the HOX family of genes, significantly enhances the proliferation, invasion and metastasis of cancer cells, and may be useful as a marker for cancer diagnosis or progression evaluation 11,12. Recently, a loss of HOXC10 expression was implicated in the development of resistance to estrogen response modulators inestrogen receptor (ER)-positive breast cancer^13^. At present, the function of HOXC10 in OSCC remains poorly understood.

In this study, we have determined that HOXC10 expression is significantly increased in human OSCC tissues, and its overexpression is significantly correlated with TNM stage, lymph node metastasis, as well as poor overall survival. Knockdown or ectopic expression assays further confirm that HOXC10 is required for migration, invasion and adhesion of OSCC cells. In addition, HOXC10 is significantly upregulated and indicates poor survival in OSCC according to the Kaplan-Meier plotter database. Bioinformatics and immunoblot analyses, and rescue experiments have been used to clarify the mechanism by which HOXC10 facilitated WNT-dependent epithelial-mesenchymal transition (EMT). Altogether, our work demonstrates that HOXC10 enhances the metastasis of human OSCC cells and may be a potential target for treatment of OSCC and a marker for prognosis of OSCC patients.

## Materials and methods

### Ethics and patient tumor sections

We used 57 primary OSCC tissue specimens from patients who had undergone surgery at the Second Xiangya Hospital of Central South University from October 2010 to December 2011 14. No patients involved in this investigation received chemotherapy prior to surgery. Clinicopathologic data of each patient are shown in Table S1. Between one and five bulk tissue samples of approximately 5 mm in size were immediately cut from the oral tissues resected by a standard surgical procedure. The tissue samples were snap frozen in liquid nitrogen and stored at -80°C until use. This study was approved by the Ethics Committee of the Second Xiangya Hospital of Central South University, and informed consent was obtained from all the patients.

### Cell lines, antibodies and reagents

The human OSCC cell lines Cal 27, FaDu, SCC25, SCC23 and SCC4 were purchased from the Shanghai Cell Bank of the Chinese Academy of Sciences and were cultured in Dulbecco's modified Eagle's medium (DMEM; Gibco, Carlsbad, CA, USA) supplemented with 10% fetal bovine serum (FBS; Gibco) and incubated at 37°C in a humidified atmosphere containing 5% CO_2_.

### Immunohistochemistry

Fixed tissues were embedded in paraffin and were sectioned at a thickness of 3μm, and immunohistochemical staining was performed with antibodies to E-cadherin (abcam, ab76055, 1:100), HOXC10 (abcam, ab153904, 1:100), N-cadherin (abcam, ab76057, 1:100), Vimentin (abcam, ab8978, 1:100), DVL2 (abcam, ab137528, 1:100) and Wnt10B (abcam, ab70816, 1:100) at 4°C overnight. The sections were mounted in a mounting medium containing glycerol (Beyotime, P0126) and visualized with a light microscope (Leica, DM6000M).

### Quantitative real-time PCR

Total RNA was isolated by using TRIzol reagent (Invitrogen, Carlsbad, CA, USA) and reverse transcribed by using the Prime Script RT reagent kit (Invitrogen, Carlsbad, CA, USA). The reaction conditions were pre-denaturation at 95 °C for 1 min, 30 cycles of denaturation at 95 °C for 30 s, annealing at 58 °C for 5 s, and extension at 72 °C for 5 s. The primer sequences for all experiments are shown in Table S2. qRT-PCR and data collection were performed with an iQ5 Real-Time PCR Detection System (Bio-Rad, USA) system. The mean fold change is shown as the natural logarithm of RQ values, and the error was estimated by evaluating the 2^-△△Ct^ equation using △△Ct plus the standard deviation and △△Ct minus the standard deviation.

### HOXC10 shRNA and stable transfections

To further analyze the role of *HOXC10* in OSCC malignancy, FaDu and SCC4 cells were transfected with HOXC10 shRNA. Cells were transfected with 1.4 ug/ml shRNA duplexes by using Lipofectamine 2000 (Invitrogen) for 48 h following the procedure recommended by the manufacturer. Control shRNA: sequence was (Forward: 5′-UUCUCCGAACGUGUCACGUDTDT-3′, Reverse: 5′-ACGUGAC ACGUUCGGAGAADTDT-3′). The human HOXC10 shRNA sequence was (Forward: 5′-TGCATGCCCTCGCAATGTAACTCCGAATTCAAGAGATTCGGAGTTACATTGCGAGGGCATGCTTTTTTC-3′, Reverse: 5′- TCGAGAAAAAAGCATGCCCTCGCAATGTAACTCCGAATCTCTTGAATTCGGAGTTACATTGCGAGGGCATGCA-3′). Control cells were transfected with a control shRNA that did not match any known human coding cDNA. Stable knockdown clones were pooled and used for experiments.

### Wound healing assay

Cells were seeded in six-well plates to 100% confluency. After serum starvation for 10 h, a wound was induced by scratching the cell cultures with a 5-μl pipette tip. Following three rinses with PBS to remove the detached cells, the adherent cells were cultured in medium without serum. Images of four random fields of each well were captured immediately and again after 3 h and 6 h using a microscope (Nikon Corporation, Tokyo, Japan) at ×10 magnification. Then, the distance between the wound edges was calculated with software from the Nikon Application Suite and the experiments were independently performed in triplicate.

### Transwell invasion assays

Transwell polycarbonate membrane inserts (6.5 mm) with 8-μm pores (Corning, Albany, NY) were embedded with 120 μg of Matrigel (BD Biosciences, San Jose, CA, USA) and 100 μg of gelatin (Sigma-Aldrich, St Louis, MO, USA) in DMEM. Either FaDu or SCC4 cells (1×10^5^ per well) in serum-free medium were added to the Matrigel-embedded inserts (the top chambers), and the inserts were placed into chambers containing 10% FBS media. After incubation for 36 h at 37°C, the remaining cells in the upper chamber were carefully removed with a cotton swab, and the cells that had invaded through the Matrigel were stained with hematoxylin, photographed and quantified.

### Soft agar assay

For the clonogenic assay, 1 × 10^3^ FaDu and SCC4 cells were plated in 35-mm culture dishes in complete DMEM. Cells were grown under these conditions for 7-10 days, and adherent separated clones were counted, as our previous study^15^.

### Bioinformatics analysis

UALCAN (http://ualcan.path.uab.edu/analysis.html), is a tool for in-depth analyses of The Cancer Genome Atlas (TCGA) data 16 , which can be utilized to predict the interaction partners of gene of interest. We used UALCAN analysis to identify the HOXC10-correlated genes in the human head and neck squamous cell carcinoma (HNSCC) datasets of TCGA, which was performed using the comprehensive set of the Database for Annotation, Visualization and Integrated Discovery (DAVID) functional annotation tools (https://david.ncifcrf.gov/) to analyze the Kyoto Encyclopedia of Genes and Genomes (KEGG) pathway 17.

### Western blot analysis

Harvested cells were lysed in RIPA buffer (Beyotime) containing a complete mini-protease inhibitor cocktail and phosphate inhibitors (Roche, Branchburg, NJ). Antibodies against human HOXC10 (abcam, ab153904, 1:1000), Wnt10B (abcam, ab70816, 1:1000), Snail (ThermoFisher, 14-9859-82, 1:1000), E-cadherin (abcam, ab76055, 1:1000), N-cadherin (abcam, ab76057, 1:1000), Vimentin (abcam, ab8978, 1:1000) were used as primary antibodies. GAPDH was used as a loading control. Each assay was performed in triplicate.

### Immunofluorescence and confocal microscopy

Cells were cultured on 10mm glass-bottom dish (Nest Biotechnology), fixed with 4% paraformaldehyde and permeabilized in 0.1% Triton X-100. After that cells were washed with PBS and blocked with 5% bovine serum albumin (BSA). The cells were incubated with primary antibodies against E-cadherin (abcam, ab76055, 1:100), N-cadherin (abcam, ab76057, 1:100), and Snail (Thermo Fisher, 14-9859-82, 1:100)) and a secondary antibody. Coverslips were mounted with the mounting medium (Vector Laboratories) containing diamidino-2-phenylindole (DAPI) and photographed under a laser scanning confocal microscope.

### *In vivo* cell growth and metastasis assay

Male athymic nude mice aged 4-weeks were purchased and housed in the animal center of Central South University. Mice were kept under pathogen-free conditions at room temperature with 12 h light/12 h dark exposure. Food and water were offered ad libitum. All operations were carried out according to the National Institutes of Health Guide for the Care and Use of Laboratory Animals. For the cancer cell growth assay, HOXC10 knockout (KO) and wild-type (*WT*) FaDu cells were transplanted into nude mice (5 nude mice per group, 2×10^6^ cells for each mouse) subcutaneously. Xenografts were measured three times a week, and the tumor volume was calculated by the formula 0.5 × L × W^2^ (L, length; W, width). After 5 weeks, mice were sacrificed, and the tumors were removed and subjected to further experiments.

### Statistical analysis

All statistical analyses were carried out using Graph-Pad Prism version 5.00 for Windows (Graph-Pad Software Inc.). Unpaired t-test was used to compare the differences in colony numbers, tumor volumes and migrated cells between the negative control group and shHOXC10 group. A one-way ANOVA followed by the post hoc Tukey test was used to analyze the differences in immunohistochemical staining. Overall survival analysis was performed using the Kaplan-Meier method. The correlation between HOXC10 expression and clinical parameters was determined using the Pearson's χ^2^ method (with the continuity correction when the number of patients was <5). The data were presented as the mean ± SEM, and statistical significance was determined as P < 0.05.

## Results

### Overexpression of HOXC10 in oral squamous cell carcinoma (OSCC) and association with poor prognosis

**Figure 1:**
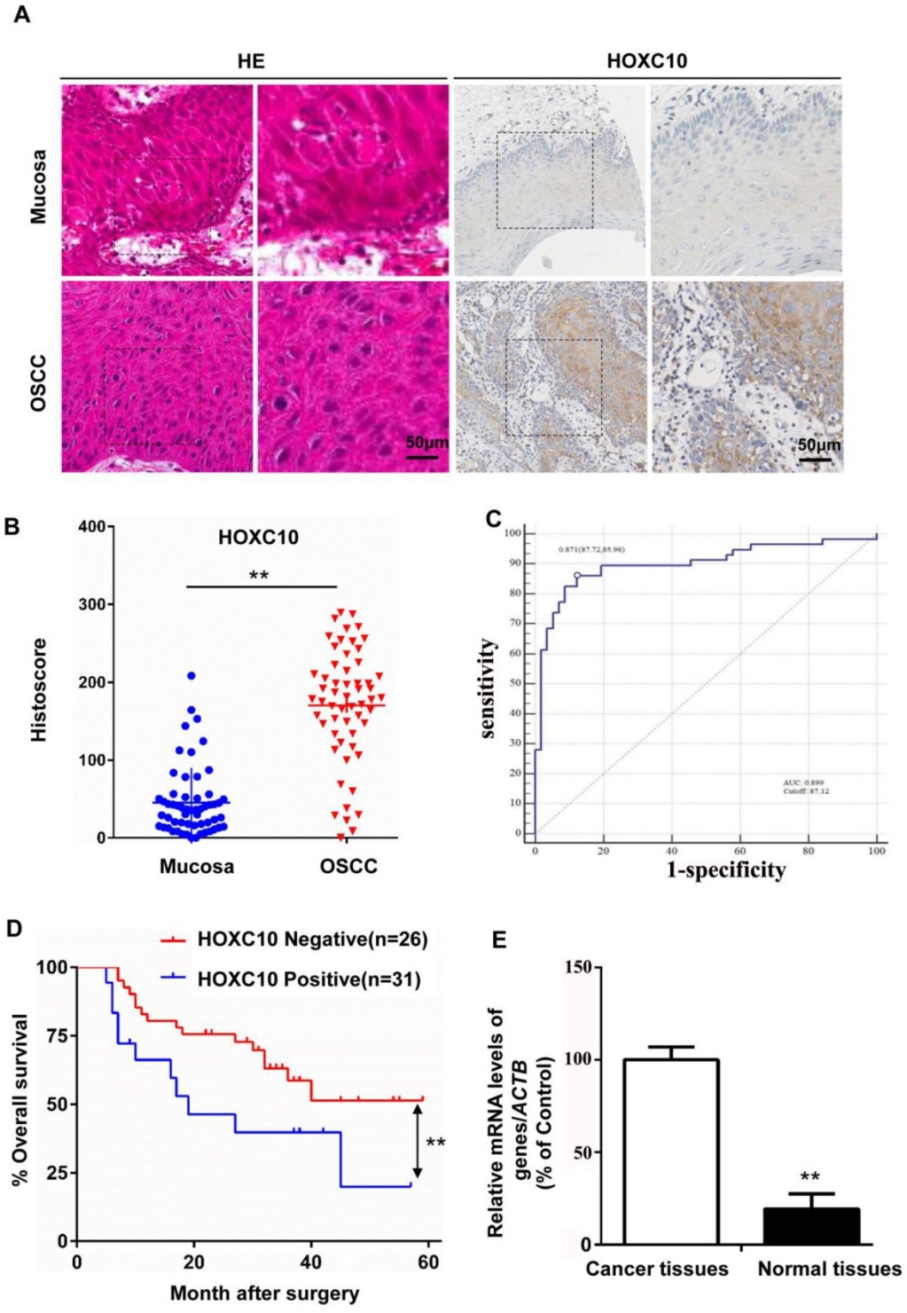
HOXC10 expression was up regulated in oral squamous cell carcinoma (OSCC) (A). Representative immunohistochemical staining of HOXC10 in human OSCC tissue compared with that in normal mucosa; scale bar: 50 μm. (B). Quantification of HOXC10 expression levels in human mucosa and OSCC tissue. (C). ROC curve analysis was adapted to determine cut-off score for dichotomizing as low expression and high expression of HOXC10. (D). Kaplan-Meier curve of overall survival in 57 patients with OSCC stratified by the expression level of HOXC10. The duration of survival was measured from the beginning of the treatment to the time of death or at 60 months. The cumulative survival for OSCC patients with positive HOXC10 expression was significantly lower than that for OSCC patients who were HOXC10-negative. (E). The relative HOXC10 mRNA level was detected by RT-PCR in OSCC patients in fresh, paired cancer and normal tissues. The data are presented as the means ± SEM. **P<0.01 versus the control group.

HOXC10 protein and mRNA levels in OSCC (n = 57) and normal mucosa samples (n = 57) were measured by immunohistochemical staining (Fig. 1B) and quantitative real-time PCR (qRT-PCR; Fig. 1E), respectively. The results showed that HOXC10 expression was increased in OSCC. The results were further confirmed with immunochemistry in the same samples (Fig. 1A). To further show the diagnosis accuracy of HOXC10, ROC analysis were also performed, AUC around 0.899 were obtained and cut-off score is 87.12 (Fig. 1C). We plotted the overall survival for HOXC10 using Kaplan-Meier curves. Based on the follow-up data of the 57 OSCC patients, we analyzed whether HOXC10 expression affected the overall survival (OS). The Kaplan-Meier survival curves showed that patients with higher HOXC10 expression had a significantly poorer 5-year OS (Fig. 1D). Moreover, the multivariate Cox regression analyses revealed that HOXC10 expression was an independent prognostic factor for poor OS. (P=0.008; Table S1). These results suggest that HOXC10 upregulation is strongly associated with poor prognosis in OSCC patients.

### HOXC10 knockdown inhibits aggressive tumor behaviors

**Figure 2:**
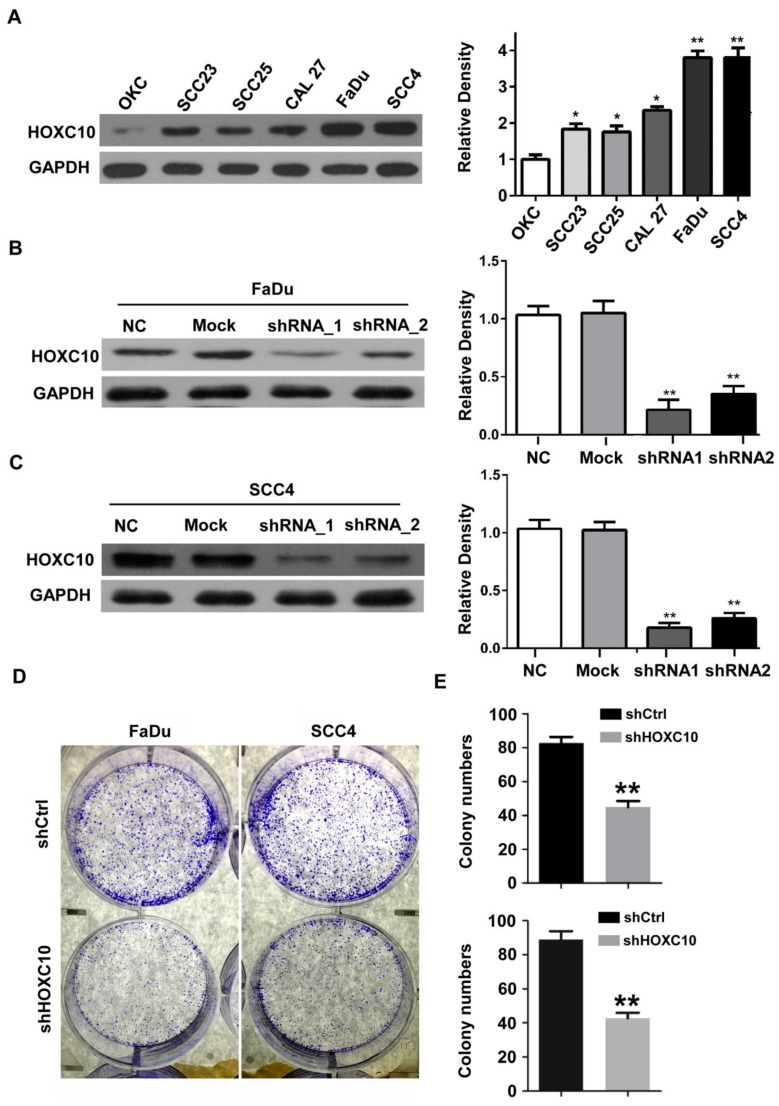
Expression analysis of HOXC10 in OSCC cell lines. (A). Western blot analysis was performed to assess the expression level of HOXC10 in OKC and OSCC cell lines; GAPDH served as a loading control. (B). Knockdown of HOXC10 by two different *shHOXC10* sequences in the FaDu cell line; GAPDH served as a loading control. (C). Knockdown of HOXC10 by two different *shHOXC10* sequences in the SCC4 cell line; GAPDH served as a loading control. The relative density data were calculated by ImageJ software. (D). Colony formation of control and shHOXC10 cells in soft agar after 14 days of culture. The data are presented as the means ± SEM. *P<0.05, **P<0.01 versus the control group. (n=6)

**Figure 3:**
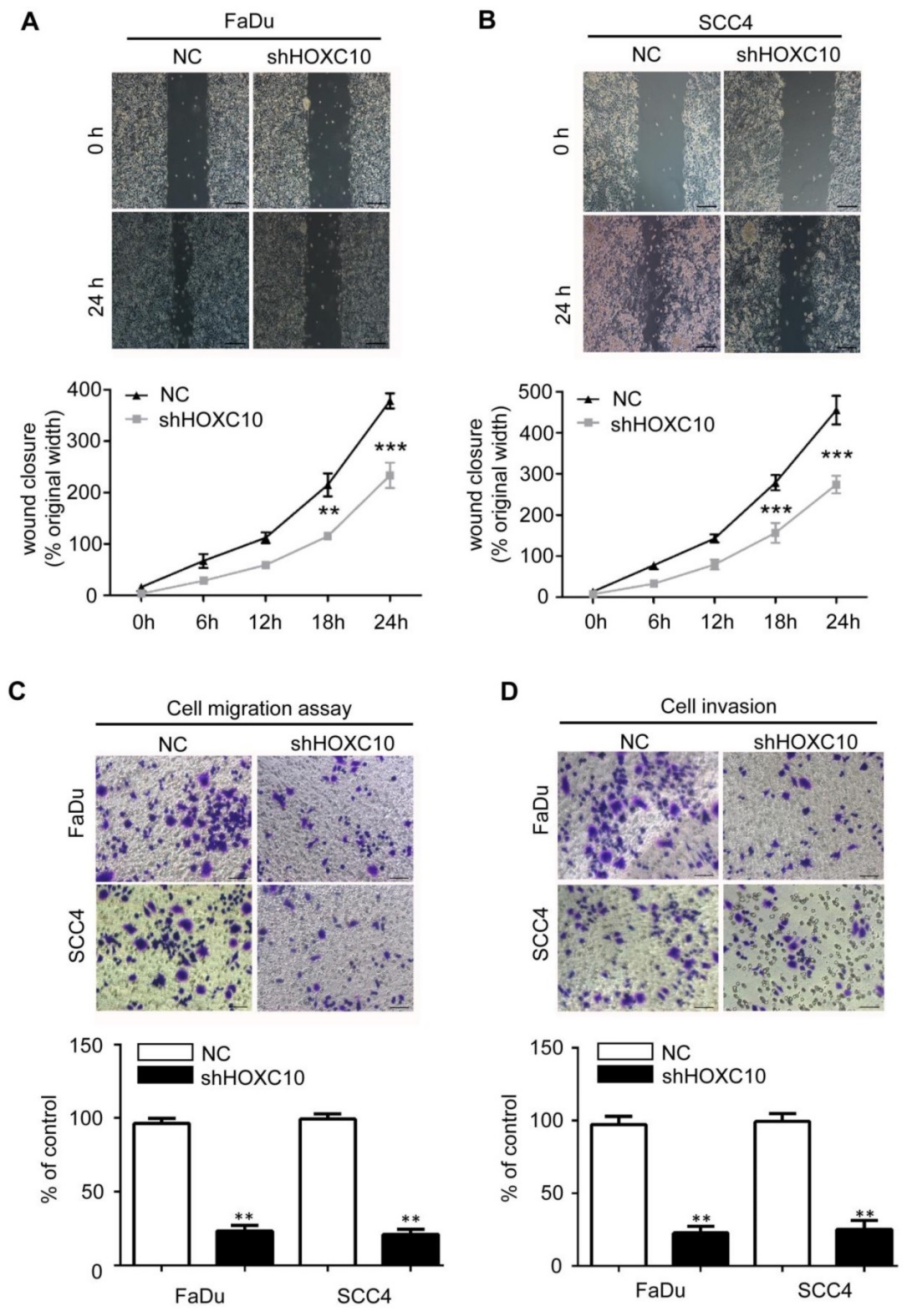
Depleting HOXC10 by RNA interference decreased OSCC cell line migration and invasion. Wound-healing assay showed that knockdown of HOXC10 suppressed cell mobility of FaDu (A) and SCC4 (B) cell lines, and the quantification of the wound closures show a statistically significant difference; scale bar: 200 μm. The transwell assay showed that the migration (C) and invasion (D) abilities of FaDu and SCC4 cells were impaired after knockdown of HOXC10 compared with those in the negative control group, and the quantification of cell numbers with the ImageJ; scale bar: 50 μm. The data are presented as the means ± SEM. *P<0.05, **P<0.01, ***P<0.001 versus the control group. (n=6).

Next, we investigated the effects of HOXC10 knock-down on invasion and/or migration of OSCC cells *in vitro.* We first detected the HOXC10 expression in 5 OSCC cell lines (Cal27, FaDu, SCC23, SCC4 and SCC25) compared with that in normal oral squamous epithelia keratinocytes (OKCs). The results (Fig. 2A) showed that HOXC10 expression was relatively higher in SCC4 and FaDu cells in comparison to several other cell lines including OKC, SCC23, SCC25 and CAL27. Therefore we chose SCC4 and FaDu cells to focus our short hairpin RNA (shRNA) experimental research on HOXC10. FaDu and SCC4 cells were infected with two different lentiviral short hairpin RNAs (shRNAs), either targeting HOXC10 or scrambled, at multiplicity of infection of 5. The effect of HOXC10 shRNAs on HOXC10 expression was evaluated with western blot, which showed that endogenous HOXC10 protein levels were efficiently knocked down by shRNA-1 and shRNA-2 (Fig. 2B and 2C). Depletion of HOXC10 prevented the colony formation of FaDu and SCC4 cells in soft-agar assay. These results indicated that the deficiency of HOXC10 specifically inhibits OSCC cell growth in monolayers and 3D cultures (Fig. 2D). Knockdown of HOXC10 notably decreased the cell mobility of FaDu (Fig. 3A) and SCC4 (Fig. 3B) cell lines, and the numbers of migratory cells were quite different between the respective control groups and the HOXC10 shRNA-treated groups for both the FaDu and SCC4 cell lines (Fig. 3C). The transwell assay also suggested that knockdown of HOXC10 decreased the transmembrane invading cell number compared with that in the negative control group, as tested in FaDu and SCC4 cell lines (Fig. 3D). These results displayed an enhanced effect of HOXC10 on OSCC cell migration and invasion.

### HOXC10 is positively correlated with Wnt signaling pathways

**Figure 4:**
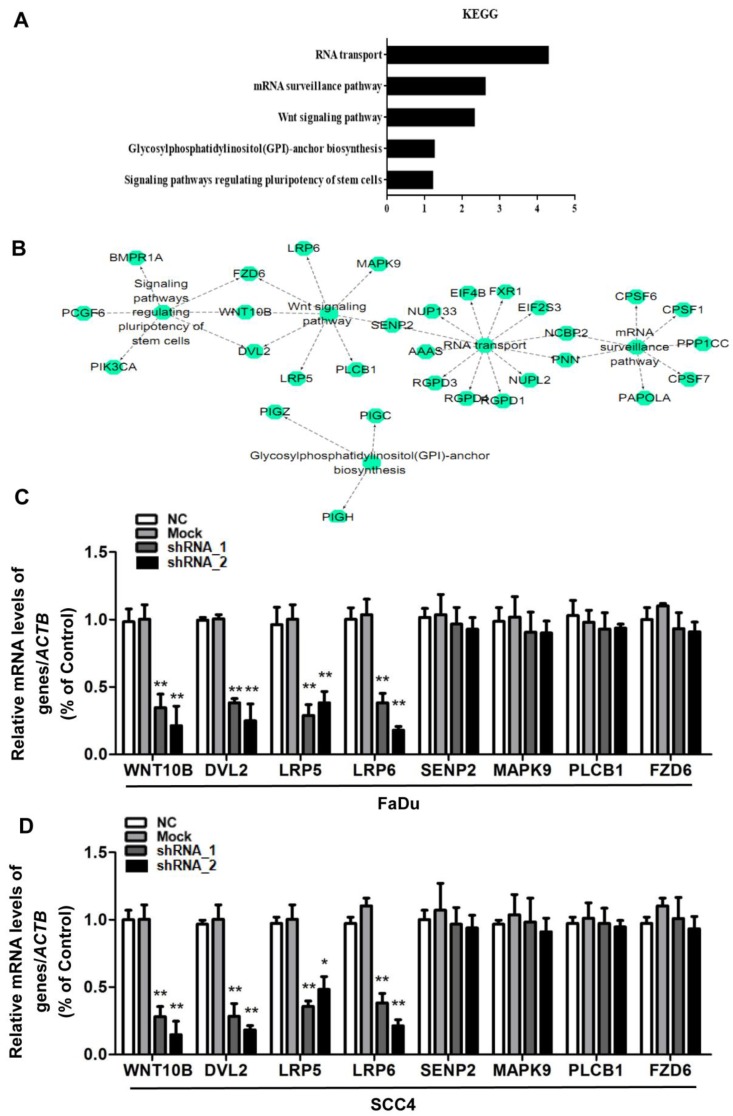
HOXC10 was positively correlated with Wnt signaling pathways. (A-B). The main enriched pathways of the HOXC10-correlated genes identified by KEGG analysis though the DAVID online database. (C). FaDu cells and (D) SCC4 cells were treated with shHOXC10, and Wnt signaling pathway-related gene expression was determined. The data are presented as the means ± SEM. **P<0.01 versus the control group.

The exact pathways that HOXC10 may regulate in human OSCC remain unclear. For an unbiased identification of the HOXC10-associated pathways, we performed UALCAN using high-throughput RNA-sequencing data of the HNSCC (OSCC is a common HNSCC) cohort of the TCGA database. UALCAN is designed to detect coordinated differences in the expression of predefined sets of functionally HOXC10-correlated genes (Table S3). Figure 4A and 4B contain mainly enriched pathways of the HOXC10-correlated genes determined by KEGG analysis though the DAVID online database. The HOXC10-correlated genes were enriched in RNA transport pathways, mRNA surveillance pathway, Wnt signaling pathway, glycosylphosphatidylinositol (GPI)-anchor biosynthesis, and signaling pathways regulating stem cell pluripotency. However, differentially HOXC10-correlated genes were mapped to KEGG pathways and enriched in 3 specific pathways including RNA transport pathways, mRNA surveillance pathway and Wnt signaling pathway, with *p*-values <0.05(Table S4).

To further confirm that HOXC10 shRNA affected Wnt signaling pathways, we also examined the Wnt signaling pathway-related gene expression. The results showed that the mRNA levels of downregulating key Wnt components, namely, Wnt10b, dishevelled segment polarity protein 2 (DVL2), LDL receptor related protein 6 (LRP6) and LDL receptor related protein 5 (LRP5) were decreased remarkably after being treated with HOXC10 shRNA in FaDu cells and SCC4 cells (Fig. 4C-D), respectively. However, other regulators of Wnt signal pathway in the KEGG analysis of Table S4 such as SUMO specific peptidase 2 (SENP2), mitogen-activated protein kinase 9 (MAPK9), phospholipase C beta 1 (PLCB1) and frizzled class receptor 6 (FZD6) were no significant changes after being treated with HOXC10 shRNA in FaDu cells and SCC4 cells (Fig. 4C-D).

### Knockdown of HOXC10 reverses the EMT phenomenon induced by Wnt

**Figure 5:**
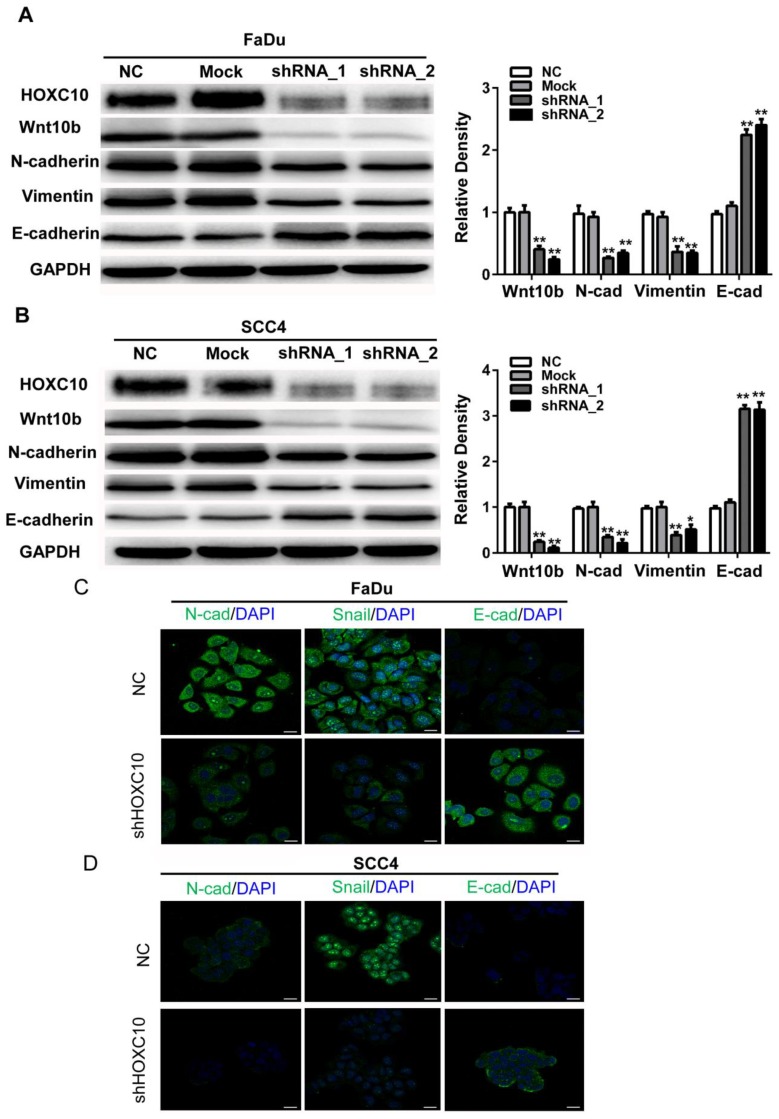
Knockdown of HOXC10 suppresses the WNT-EMT process in OSCC cell lines. (A). FaDu cells and (B) SCC4 cells were treated with shHOXC10, and Wnt10B, N-cadherin, E-cadherin, and Vimentin levels were determined. GAPDH served as an internal standard for protein loading. (C). FaDu cells and (D) SCC4 cells were treated with negative control (NC) and shHOXC10; representative immunofluorescence is shown, and fluorescence of N-Cadherin, Snail and E-cadherin was quantified; scale bar: 20 μm. The data are presented as the means ± SEM. **P<0.01 versus the control group.

Accumulating evidence suggests that Wnt signaling contributes to EMT induction and regulates asymmetric cell-fate decisions in human mammary stem cells. Here, we found that Wnt10B was markedly suppressed in shHOXC10-Fadu and shHOXC10-SCC4 cells (Fig. 5A). To further confirm the relationship between HOXC10 and EMT progression, we employed shRNA to knock down HOXC10 and detect alterations in the EMT as indicated by putative EMT markers *in vitro* via western blotting. After knockdown of HOXC10, the expression levels of E-cadherin were upregulated, while N-cadherin and Vimentin were downregulated in each of the HOXC10-silenced groups compared with that in the negative control groups in FaDu cells (Figure 5A) and SCC4 cells (Fig. 5B). Furthermore, we detected the morphological expression of the cell lines with immunofluorescence. Similar results were observed in SCC4 cell lines: Snail was decreased while E-cadherin was increased after knockdown of HOXC10 (Fig. 5C). These results suggested that HOXC10 may reverse the EMT phenomenon induced by Wnt10B.

### Knockdown of HOXC10 blocks tumor growth, invasion and metastasis of OSCC *in vivo*

**Figure 6:**
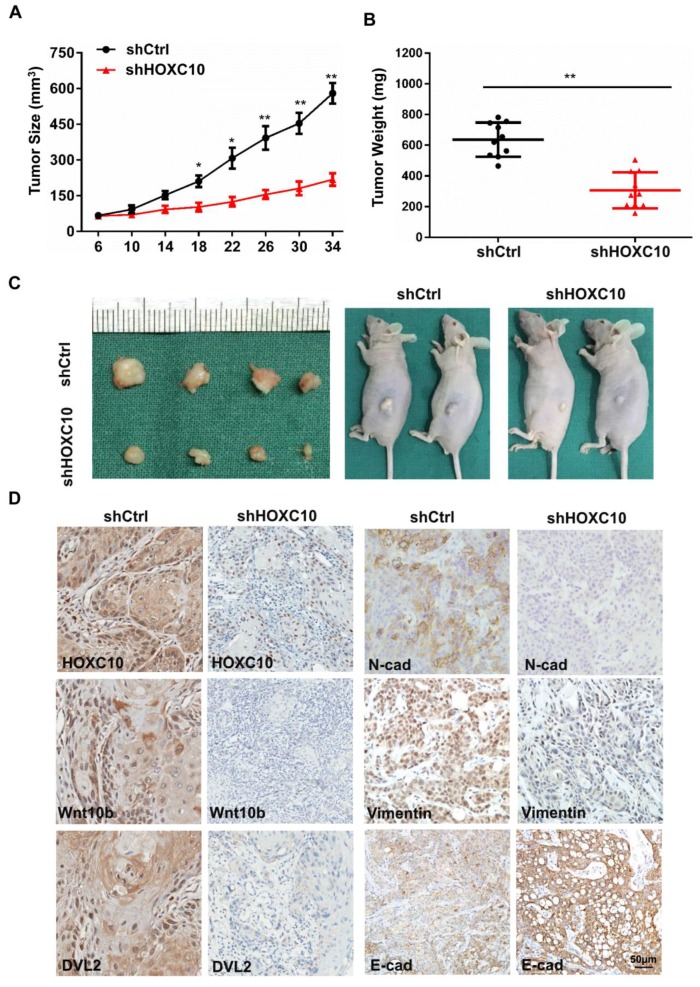
Knockdown of HOXC10 blocks tumor growth, invasion and metastasis of OSCC *in vivo*. (A). Tumor growth curve for shHOXC10 and control mice. (B). The tumor volume and weight were measured. (C). Dissected tumors were photographed. (D). Representative images of immunohistochemical analysis of HOXC10, Wnt10B, DVL2, E-cadherin, N-cadherin and Vimentin in tumors; scale bar: 50 μm. The data are presented as the means ± SEM. *P<0.05, **P<0.01 versus the control group.

Based on our *in vitro* findings, we investigated whether HOXC10 could promote invasion and metastasis of OSCC *in vivo*. A xenograft tumor model was established via subcutaneous injection of FaDu cells into nude mice. Suppression of HOXC10 significantly inhibited tumor growth, and reduced tumor volume (P < 0.001) (Fig. 6A) and tumor weight (P < 0.001) (Fig. 6B and 6C). Immunohistochemistry confirmed that HOXC10 knockdown resulted in suppression of N-cadherin and Vimentin, increased E-cadherin expression and partial suppression of Wnt10B and DVL2 (Fig. 6C). These findings suggest that knockdown of HOXC10 could inhibit EMT progression and that targeting HOXC10 can reduce tumor growth, invasion and metastasis of OSCC cells* in vivo*.

### HOXC10 is positively correlated with Wnt-EMT signaling pathways in OSCC patients

**Figure 7:**
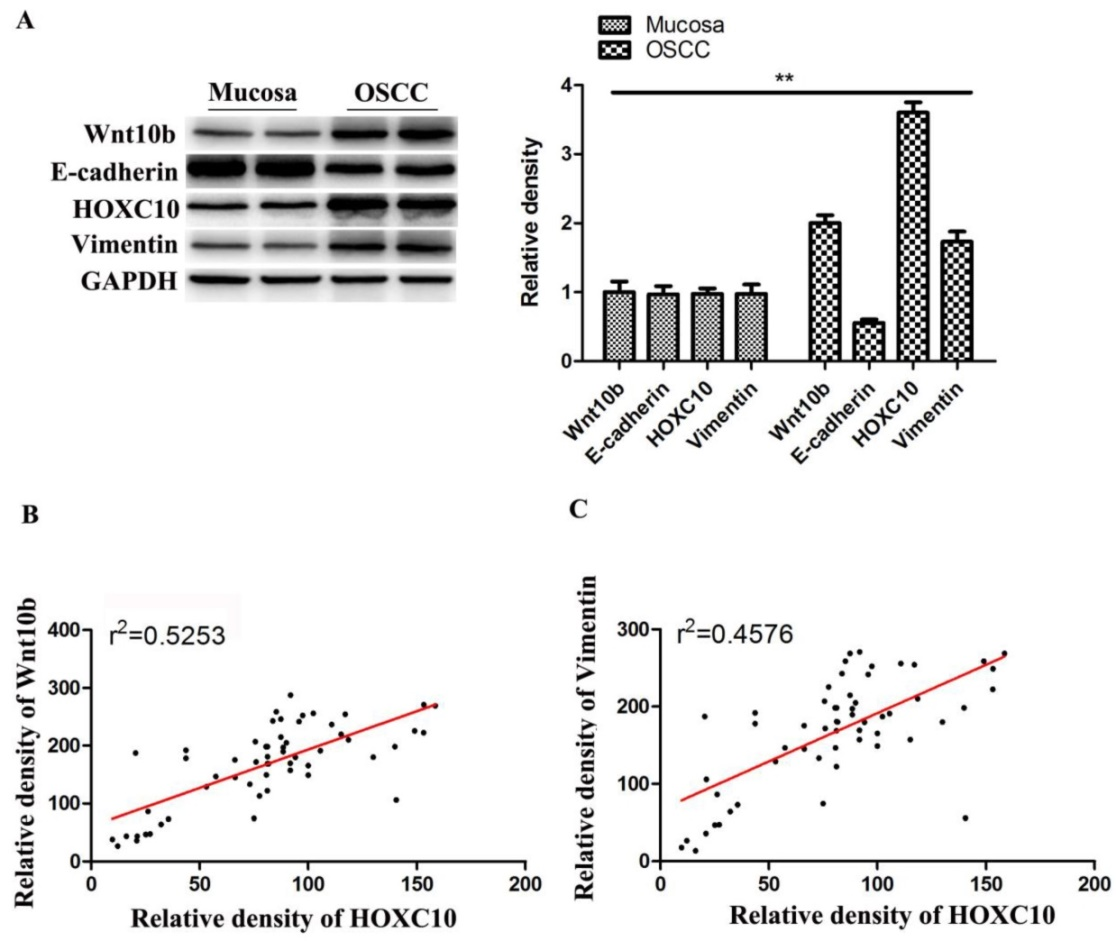
HOXC10 is positively correlated with Wnt-EMT signaling pathways in OSCC patients. (A). The protein levels of Wnt10b, E-cadherin and Vimentin were determined. (B). Correlation of HOXC10 and Wnt10b protein levels in OSCC samples. (C). Correlation of HOXC10 and Vimentin protein levels in OSCC samples. **P<0.01, versus the normal mucosa group;

Western blot analysis showed that Wnt10b and Vimentin expressions were upregulated, while E-cadherin were downregulated in OSCC tissues samples than in normal mucosa samples (Fig.7A). Notably, HOXC10 expression and Wnt10b protein levels showed a significant positive correlation in OSCC tissues (r^2^= 0.5253, P<0.0001) (Fig. 7B**)**. Furthermore, HOXC10 expression and Vimentin protein levels were also positively correlated in patients with OSCC (r^2^ = 0.4576, P<0.0001) (Fig. 7C**)**.

## Discussion

HOXC10 plays important roles in many types of cancer, and its upregulation is also found in human gastric cancer 18, cervical squamous cell carcinomas 19, and breast cancer 20. In addition, HOXC10 upregulation is associated with lymph node metastases and chemotherapy resistance in breast cancer 12, increased invasiveness in cervical squamous cell carcinomas and short survival in human lung adenocarcinoma 11,21. These previous studies are fully consistent with our current findings that HOXC10 expression is increased in OSCC and is associated with high histological grade. In the present study, we conducted* in vitro* and *in vivo* experiments to determine the clinical significance of HOXC10 in OSCC and further characterize the molecular mechanisms by which this gene contributes to disease pathogenesis.

Overexpression of HOXC10 frequently detected in various cancers 12,18,22, promotes the growth and metastasis of malignant cells, and has a negative impact on the clinical outcome. In the present study, we identified by IHC staining a significant increase in HOXC10 protein level in OSCC tissues compared with the normal oral mucosa. This result, which was in accordance with the studies on many other cancers, emphasized the oncogenic role of HOXC10 in OSCC. Recent studies showed that abnormal HOXC10 expression contributed to the survival of cancer cells 11,23. In breast cancer, elevated HOXC10 expression helped malignant cells to survive under serum-depleted or hypoxic conditions 23. Meanwhile, HOXC10 promotes gastric cancer cell invasion and migration via regulation of the Nuclear factor-κB (NF-κB) pathway 18. Migration and invasion are crucial processes for tumor cell circulation and establishment of distant metastasis 22. Several investigations into tumor progression have indicated the pro-metastasis role of HOXC10 20,22. In the present study, we found a significantly higher HOXC10 expression in high-metastatic OSCC cell lines compared with the OKC cell lines. Furthermore, inhibition of HOXC10 significantly attenuated the migration ability of OSCC cells. These findings implied an indispensable role of HOXC10 in metastasis of OSCC cells. However, the underlying complex mechanism remains to be elucidated. Taken together, our findings indicate that HOXC10 may promote tumor progression by enhancing cell proliferation and metastasis in OSCC.

The EMT process is initiated by signaling pathways that respond to extracellular cues, among which WNT plays a predominant role. In breast cancer, Wnt10B was found to be upregulated to promote metastasis and interfere with the clinical outcome 24. In pancreatic cancer, Wnt10B, combined with β-catenin, enhanced the EMT and contributed to cancer dissemination 25. Activation of Wnt10B/β-catenin axis was significantly associated with poor survival of patients with pancreatic cancer. These studies emphasized the predominate role of Wnt10B in inducing and maintaining EMT. A growing number of studies have demonstrated that tumor cells that undergo EMT acquire better survival and stronger metastatic capabilities. It has been reported that Wnt10B potently contributed to the migration and invasion of OSCC cells 26. Indeed, an enhanced migration ability of OSCC cells was induced by Wnt10B in our present study, but it was significantly reversed by HOXC10 knockdown. HOXC10 silencing reduced the N-cadherin, Vimentin and Wnt10B levels, but upregulated the E-cadherin level in OSCC cells. These observations were partially in accordance with other research. In summary, our data suggest that HOXC10 may partially disrupt the metastasis of OSCC cells by decreasing EMT.

In summary, our study shows that HOXC10 is overexpressed in OSCC cells and tumor tissues. Knockdown of HOXC10 inhibits cell proliferation, suppresses the EMT and inhibits OSCC cell invasion and migration via Wnt signaling. Thus, HOXC10 may be a potential prognostic biomarker, and a therapeutic target in OSCC. Our study thus sheds new light on the pro-tumorigenic effects of HOXC10 in OSCC.

## Supplementary Material

Table S1.
