# Supplementary material for: HOXC10 promotes migration and invasion via the WNT-EMT signaling pathway in oral squamous cell carcinoma
Source: J Cancer. 2019 Jul 25;10(19):4540–51. doi: 10.7150/jca.30645 (PMC6746115; doi:10.7150/jca.30645)
Supplement: Supplementary file 1 — Table S1. [file jcav10p4540s1.pdf]

Table S1: Statistical Analyses of Factors Associated With Survival in OSCC Patients with the Multivariate Cox Proportional Hazards Model.

Variables	Overall survival		*P* value
	RR	95% CI	
HOXC10 expression			
Positive VS negative	**2.551**	**1.167-4.319**	**0.005**
Gender			
Male VS female	0.721	0.327-2.120	0.713
Age			
＜60 VS ≥60	1.413	0.899-2.553	0.912
Smoking			
Yes VS no	0.821	0.335-2.117	0.663
Drinking			
Yes VS no	0.997	0.661-3.213	0.341
Tumor stage			
3-4 VS 1-2	**3.117**	**1.421-4.871**	**0.007**
Lymph node metastasis			
+ VS -	**3.237**	**1.378-4.691**	**0.000**
Clinical stage			
3-4 VS 1-2	**2.169**	**1.214-4.641**	**0.000**
Histological type			
Poor VS well-moderate	1.217	0.764-1.214	0.764

CI, confidence interval.

Table S2: Sequences of primers used in quantitative RT-PCR

Target gene	Primer	Nucleotide sequence
HOXC10	F<br>R	5'- -ACATCTGGAATCGCCTCAGC-3'<br>5'- GGCTCTGCTCCGTCTTGATT-3'
WNT10B	F<br>R	5'- CATCCAGGCACGAATGCGA-3'<br>5'- CGGTTGTGGGTATCAATGAAGA-3'
DVL2	F<br>R	5'- GAGGAAGAGACTCCCTACCTG-3'<br>5'- CGGGCGTTGTCATCTGAAAT-3'
SENP2	F<br>R	5'- GGCTGGTTAGGATTCTCGGC-3'<br>5'- GGCAGCATTGTAGAGACTGTTTT-3'
LRP6	F<br>R	5'- TTTATGCAAACAGACGGGACTT-3'<br>5'- GCCTCCAACTACAATCGTAGC-3'
MAPK9	F<br>R	5'-GAAACTAAGCCGTCCTTTTCAGA-3'<br>5' TCCAGCTCCATGTGAATAACCT-3'
PLCB1	F<br>R	5'- GGACTGACCCTCAGGGATTTT-3'<br>5'- AAGCCACGAGATTCAAATGGG-3'
FZD6	F<br>R	5'- ATGGCCTACAACATGACGTTT-3'<br>5'- GTTTACGACAAGGTGGAACCA-3'
LRP5	F<br>R	5'- ACTCGCTGTGAGGAGGACAAT-3'<br>5'- GGCAGGCGCATGTGTAGAA-3'
ACTB	F<br>R	5'-CATGTACGTTGCTATCCAGGC-3'<br>5'-CTCCTTAATGTCACGCACGAT-3'

Table S3. UALCAN is designed to detect coordinated differences in the expression of predefined sets of functionally HOXC10-correlated genes.

GENES	Pearson CC
HOXC11	0.54
HOXC9	0.49
HOXC6	0.49
LOC646762	0.48
HOXC4	0.48
LRRC37B2	0.46
LOC400043	0.44
NBPF15	0.43
LOC200030	0.43
WHAMML1	0.43
HOXC8	0.43
CCDC112	0.41
PRKCSH	0.41
ZNF354B	0.41
RGPD1	0.41
NBPF9	0.41
PELI2	0.41
C1orf96	0.4
CTPS2	0.4
RIC8B	0.4
BMPR1A	0.39
ZNF642	0.39
KCNMB3	0.39
TSPYL5	0.39

ZNF639	0.39
CEP290	0.38
RFXAP	0.38
CXorf23	0.38
C19orf44	0.38
SLC39A10	0.38
KIF3A	0.38
HOXA10	0.38
ANGEL2	0.38
RNF216	0.37
CAMLG	0.37
MORN4	0.37
UVRAG	0.37
MAPK9	0.37
HECTD2	0.37
USPL1	0.37
LETMD1	0.36
LASS2	0.36
ABCB10	0.36
ZNF37B	0.36
ZNF26	0.36
PDE4A	0.36
JAZF1	0.36
RBMX	0.36
LRRC37B	0.36
USP27X	0.36

LOC144438	0.36
TRAF5	0.36
ZDHHC17	0.36
KLHL15	0.36
DDX17	0.36
TTC14	0.36
MOSC2	0.36
CPSF6	0.36
FLVCR1	0.36
TTLL4	0.35
RGPD6	0.35
SLC22A5	0.35
BIVM	0.35
C5orf25	0.35
POGZ	0.35
TMED8	0.35
TRIM52	0.35
C7orf26	0.35
C1orf112	0.35
ZMYM5	0.35
MAGEF1	0.35
DZIP1	0.35
PSPC1	0.35
ZNF12	0.35
BBS5	0.35
DNAH14	0.35

ZNF333	0.35
DBF4B	0.35
CSRNP2	0.35
HNRPLL	0.35
EIF2AK1	0.35
ZNF594	0.34
SMARCD1	0.34
CLK2	0.34
FXR1	0.34
LPHN1	0.34
NCBP2	0.34
POTEF	0.34
PHKA2	0.34
TIGD1	0.34
SPATS2	0.34
WDR67	0.34
GPR89A	0.34
TIAL1	0.34
ZNF815	0.34
ZNF22	0.34
HMGB1	0.34
KBTBD6	0.34
NAA16	0.34
TIGD7	0.34
ZNF740	0.34
ZNF514	0.34

DTX3	0.34
GON4L	0.34
NVL	0.34
ZBTB10	0.34
AZIN1	0.34
EIF5A2	0.34
C7orf31	0.34
MDM4	0.34
DVL2	0.34
NHLRC3	0.34
LOC339047	0.34
KIAA0907	0.34
NEK3	0.34
ALS2	0.34
PCGF6	0.34
SARM1	0.34
C14orf101	0.34
CENPJ	0.33
MBTPS2	0.33
WDR83	0.33
DFFB	0.33
TCTN1	0.33
SCML1	0.33
TTC21B	0.33
C10orf137	0.33
RAB11FIP2	0.33

CLK2P	0.33
ALMS1	0.33
USP30	0.33
SENP6	0.33
CHRNA5	0.33
ZNF275	0.33
TARBP1	0.33
LOC646214	0.33
SLC25A36	0.33
RBM39	0.33
ACTR6	0.33
ANUBL1	0.33
ZNF558	0.33
BRAP	0.33
HOXA4	0.33
PRDM5	0.33
MAGEE1	0.33
CREBZF	0.33
PIGZ	0.33
MTA1	0.33
PMS2CL	0.33
IRF2BP1	0.33
ABI2	0.33
HOXD13	0.33
BOD1	0.33
LOC91316	0.33

ESCO1	0.33
SIRT1	0.33
SLC39A1	0.33
LSM11	0.33
GDPD5	0.32
USP42	0.32
UIMC1	0.32
WNT10B	0.32
DDX50	0.32
HNRNPH1	0.32
TCTE3	0.32
LBR	0.32
TBC1D12	0.32
TUB	0.32
HMGN4	0.32
C6orf48	0.32
NUPL2	0.32
TMEM136	0.32
RFC3	0.32
ZNF343	0.32
SEPT7P2	0.32
CDK20	0.32
DMWD	0.32
KATNAL1	0.32
MPP2	0.32
ADO	0.32

VMAC	0.32
ZBTB39	0.32
TSNARE1	0.32
MLH3	0.32
ATXN2	0.32
PIGH	0.32
POLR3C	0.32
SHROOM1	0.32
NEO1	0.32
LIN9	0.32
LOC285074	0.32
CHPT1	0.32
CHD6	0.32
DNAJC14	0.32
GGT7	0.32
SFRS7	0.32
MTR	0.32
CCNJ	0.32
MCM8	0.32
DHX40	0.32
MPHOSPH8	0.32
KCNJ14	0.32
RGPD4	0.32
NCRNA00183	0.32
NFYB	0.32
ZNF195	0.32

WDR35	0.32
PNN	0.32
ZNF169	0.32
CYB5D1	0.32
FAM149A	0.32
NEDD1	0.32
ZNF621	0.32
TROVE2	0.32
GATAD2B	0.32
EIF2C4	0.32
PECR	0.31
CCNB1IP1	0.31
ZNF121	0.31
RGPD3	0.31
SENP5	0.31
TTC5	0.31
TMEM97	0.31
ACP6	0.31
TUG1	0.31
C8orf51	0.31
CPSF7	0.31
BBS10	0.31
C10orf35	0.31
PARP1	0.31
FNBP4	0.31
C14orf4	0.31

KHDC1	0.31
SLC26A10	0.31
ZNF187	0.31
ZNF136	0.31
C6orf170	0.31
AAAS	0.31
NCOA1	0.31
AKAP8	0.31
CCDC126	0.31
KCTD18	0.31
EIF2S3	0.31
TCTN2	0.31
MOAP1	0.31
RPS6KC1	0.31
ANKRD36B	0.31
BAHCC1	0.31
NAP1L1	0.31
LUC7L3	0.31
PIGC	0.31
CEP135	0.31
MAN2A2	0.31
LOC100133991	0.31
ACBD6	0.31
ZNF653	0.31
GXYLT1	0.31
ZBTB41	0.31

AHCTF1	0.31
RAVER1	0.31
ZMYM2	0.31
RNF41	0.31
C3orf33	0.31
TGDS	0.31
ANAPC5	0.31
RAD51L3	0.31
ARID4B	0.31
G2E3	0.31
DNAL1	0.31
RRP15	0.31
RCOR3	0.31
RGAG4	0.31
BCOR	0.31
EIF4B	0.31
PLCB1	0.31
SENP2	0.31
ZCCHC8	0.31
ZNF84	0.31
PAN3	0.31
DDHD1	0.31
FOXF2	0.31
PCMTD2	0.31
LASS5	0.31
SMEK1	0.31

LOC100132287	0.31
TTC31	0.31
PIK3CA	0.31
PAPOLA	0.31
ZNF182	0.31
EP400	0.31
FAM189B	0.31
RUNDC1	0.31
SR140	0.31
MAML1	0.31
DCLRE1A	0.31
EPM2AIP1	0.31
HNRNPA1L2	0.31
ZBED5	0.31
PPPDE1	0.31
ARL10	0.31
ALOX12P2	0.31
TMEM194A	0.31
SGOL2	0.31
IPO9	0.3
SFRS6	0.3
C19orf2	0.3
FAM161A	0.3
C1orf107	0.3
FAM156A	0.3
CACNB3	0.3

DNASE2	0.3
KIAA0495	0.3
PATZ1	0.3
RBMS3	0.3
FZD6	0.3
STX16	0.3
ZNF678	0.3
PAK2	0.3
KLHL24	0.3
ARNT2	0.3
FADS1	0.3
OPA1	0.3
ZNF107	0.3
TGIF2	0.3
CHD1L	0.3
MIPEP	0.3
PHC1	0.3
TBCE	0.3
ROBO3	0.3
PCBP2	0.3
LRP6	0.3
MEST	0.3
COX11	0.3
TMX4	0.3
LOC642852	0.3
KIAA0753	0.3

ZNF317	0.3
IFT88	0.3
NEURL4	0.3
KBTBD2	0.3
CDADC1	0.3
TSPYL2	0.3
SFRS8	0.3
SLC5A6	0.3
BCORL1	0.3
NPTXR	0.3
ZNF711	0.3
DNA2	0.3
CNTROB	0.3
C2orf56	0.3
SNHG1	0.3
TRIM3	0.3
SFRS16	0.3
MEGF8	0.3
B3GALTL	0.3
INTS3	0.3
CPSF1	0.3
ZNF346	0.3
PLEKHA8	0.3
POFUT1	0.3
ZNF791	0.3
CCDC41	0.3

GORAB	0.3
TTC13	0.3
R3HDM2	0.3
NUP133	0.3
SYNJ2BP	0.3
TFCP2	0.3
HIBADH	0.3
ZNF251	0.3
C5orf24	0.3
ZNF669	0.3
RPAP3	0.3
ARHGEF11	0.3
RNF219	0.3
PRAME	0.3
TRIM59	0.3
NUDT17	0.3
TRA2B	0.3
KIAA1715	0.3
CBFA2T2	0.3
AURKAPS1	0.3
GANAB	0.3
LRP5	0.3
DLEU2	0.3
HHEX	0.3
RNASEH2B	0.3
PTPDC1	0.3

DSG2	0.3
FAM169A	0.3
LOC400927	0.3
ERCC3	0.3
ZNF14	0.3
PRKD1	0.3
FGD1	0.3
UBR7	0.3
UBXN2A	0.3
C5orf42	0.3
SFRS14	0.3
SV2A	0.3
ADSS	0.3
RNF19A	0.3
C13orf23	0.3
C1orf55	0.3
ZNF354A	0.3
C10orf28	0.3
RABL2B	0.3
C2orf3	0.3
PPM1D	0.3
RTN4R	0.3
PRKD3	0.3
NUPL1	0.3
PPP1CC	0.3
CRTC2	0.3

ZBTB8A	0.3
CCDC150	0.3
LOC728190	0.3
FAM82A1	0.3

Table S4: KEGG pathways associated with the correlated genes.

Term	Count	Genes	*P* value
hsa03013:RNA transport	12	NCBP2, EIF4B, SENP2, NUP133, AAAS, RGPD1, RGPD4, RGPD3, EIF2S3, NUPL2, PNN, FXR1	4.95E-05
hsa03015:mRNA surveillance pathway	7	NCBP2, PAPOLA, CPSF7, CPSF6, PPP1CC, CPSF1, PNN	0.002384187
hsa04310:Wnt signaling pathway	8	DVL2, SENP2, WNT10B, LRP6, MAPK9, PLCB1, LRP5, FZD6	0.004620306
hsa00563:Glycosylphosphatidylinositol(GPI)-anchor biosynthesis	3	PIGZ, PIGH, PIGC	0.05377096
hsa04550:Signaling pathways regulating pluripotency of stem cells	6	DVL2, WNT10B, PCGF6, PIK3CA, BMPR1A, FZD6	0.059469658
